# What Generates Attention to Health in Trade Policy-Making? Lessons From Success in Tobacco Control and Access to Medicines: A Qualitative Study of Australia and the (Comprehensive and Progressive) Trans-Pacific Partnership

**DOI:** 10.34172/ijhpm.2020.80

**Published:** 2020-06-07

**Authors:** Belinda Townsend, Sharon Friel, Ashley Schram, Fran Baum, Ronald Labonté

**Affiliations:** ^1^School of Regulation and Global Governance, College of Asia and the Pacific, Australian National University, Canberra, ACT, Australia.; ^2^Southgate Institute for Health, Society and Equity, Department of Public Health, Flinders University, Adelaide, SA, Australia.; ^3^Institute of Population Health, University of Ottawa, Ottawa, ON, Canada.

**Keywords:** Agenda-Setting, Trade Policy, Governance, Non-communicable Disease, Health Policy

## Abstract

**Background:** Despite greater attention to the nexus between trade and investment agreements and their potential impacts on public health, less is known regarding the political and governance conditions that enable or constrain attention to health issues on government trade agendas. Drawing on interviews with key stakeholders in the Australian trade domain, this article provides novel insights from policy actors into the range of factors that can enable or constrain attention to health in trade negotiations.

**Methods:** A qualitative case study was chosen focused on Australia’s participation in the Trans-Pacific Partnership (TPP) negotiations and the domestic agenda-setting processes that shaped the government’s negotiating mandate. Process tracing via document analysis of media reporting, parliamentary records and government inquiries identified key events during Australia’s participation in the TPP negotiations. Semi-structured interviews were undertaken with 25 key government and non-government policy actors including Federal politicians, public servants, representatives from public interest nongovernment organisations and industry associations, and academic experts.

**Results:** Interviews revealed that domestic concerns for protecting regulatory space for access to generic medicines and tobacco control emerged onto the Australian government’s trade agenda. This contrasted with other health issues like alcohol control and nutrition and food systems that did not appear to receive attention. The analysis suggests sixteen key factors that shaped attention to these different health issues, including the strength of exporter interests; extent of political will of Trade and Health Ministers; framing of health issues; support within the major political parties; exogenous influencing events; public support; the strength of available evidence and the presence of existing domestic legislation and international treaties, among others.

**Conclusion:** These findings aid understanding of the factors that can enable or constrain attention to health issues on government trade agendas, and offer insights for potential pathways to elevate greater attention to health in future. They provide a suite of conditions that appear to shape attention to health outside the biomedical health domain for further research in the commercial determinants of health.

## Introduction

The need to address the nexus between trade agreements and their potential health impacts has been a topic of global concern for at least the past twenty years.^1^ Over the last decade, public health scholars have researched the causal pathways by which trade and investment agreements have shaped the social and commercial determinants of health.^2-4^ Analyses have highlighted both the potential positive impacts of trade (for example through greater access to health promoting goods and services), as well as the potential negative impacts including constraints on governments’ ability to regulate and the liberalisation of trade in health-harmful commodities.^5-9^ Indeed, the global rise in non-communicable diseases (NCDs) as “one of the major challenges for development in the 21st century”^10^ is due to a rise in NCD risk factors including tobacco use, harmful use of alcohol, poor physical activity and poor nutrition.^11^ Trade agreements have facilitated these NCD risk factors by increasing the volume of health harmful commodity imports, as well as local production, manufacturing and distribution of these products through goods and services liberalisation, provisions that reduce tariffs (ie, border taxes) and the elimination of restrictions on foreign direct investment.^12^ Greater influence within regulatory environments afforded to corporations through harmonisation and investor-state dispute settlement (ISDS) has also interfered with efforts to regulate the sale of these harmful commodities.^13^ Furthermore, expansive intellectual property rights in trade agreements, which include extending pharmaceutical monopolies, can negatively affect access to treatment by keeping medicine prices higher for longer.^14^ These analyses have led to calls by public health experts, advocacy groups, and intergovernmental bodies for greater attention to the potential health consequences of trade negotiations at the national level and in multilateral trade forums.^15,16^ Public health scholars and policy-makers, however, continue to lament that little in practice has been achieved, and that public health remains largely on the periphery of trade policy-making.

What remains understudied are the political and governance conditions that enable or constrain attention to health issues on government trade agendas. Key trade governance challenges noted in the literature include issues of transparency, accountability, participation, integrity and capacity.^17^ Analysis of framing in trade policy has highlighted the power of a dominant neoliberal market-oriented discourse that privileges exporter interests and market liberalisation.^18^ This dominant framing aligns with the interests of exporters including multinational pharmaceutical companies and ultra-processed food and alcohol exporters.^19-21^ Studies have also highlighted the ways in which public health advocates attempt to influence trade negotiations and the different claims to authority and legitimacy used by non-state actors to attempt to influence negotiations.^22^ Research on non-state actors engagement in trade negotiations note power asymmetries in access to informal and formal policy processes between industry and public health actors.^23,24^How actors, ideas, political contexts and health issue characteristics converge to enable or constrain attention to NCD risk factors in trade policy is less well understood, and is the focus of this paper.

 The paper reports on a study of Australia’s participation in the Trans-Pacific Partnership (TPP) agreement as a case study for investigating the factors that enable or constrain health in trade negotiations. It discusses results from key informant interviews with policy-makers intimately involved in the trade policy domain in Australia. We start with a brief description of the TPP and of our theoretical approach. After describing the methods, we explore in the results the key factors shaping attention or neglect or health under key themes emerging from the interviews, and share implications of our findings for future research.

###  The Trans-Pacific Partnership Agreement

 The TPP agreement was a mega-regional trade agreement negotiated between 2008 and 2015, led by the United States, and comprising a mix of low- and high-income countries. The TPP was part of a new generation of preferential trade agreements with a wide negotiating agenda beyond traditional trade rules on tariffs, with new ‘behind the border’ measures in areas such as services, investment, regulatory coherence, intellectual property, among others. Australia joined the negotiations in October 2008 and signed the agreement in late 2015. In 2017, the United States withdrew from the agreement and remaining parties renegotiated the agreement, suspending some rules, and renaming it the Comprehensive and Progressive TPP.

While the agreement was negotiated behind closed doors with no public access to the text until it was signed, public health expressed concerns with a range of probable negotiating issues. These issues, based on past analyses of trade treaties, included potential impacts on access to medicines, the need to protect space for government regulation, the liberalisation of unhealthy and harmful products, potential impacts for public health from services liberalisation, and impacts on workers’ rights and environment regulation.^25,26^ In 2015, the final year of the negotiations and based on information leaked or by then publically available, a group of academics and public health associations in Australia released a health impact assessment of the proposed agreement focused on the potential impacts for access to medicines, tobacco control, alcohol control, and food and nutrition.^27^ The final text suggested that some of these public health concerns had influenced the agenda. In particular, public health advocates noted specific provisions for tobacco control measures in the investment chapter, and less stringent pharmaceutical intellectual property measures than initially sought by the United States. Taking this outcome as the starting point, we were interested in understanding (*a*) what attention public health issues received in Australia’s negotiating mandate, and (*b*) what factors shaped attention or neglect of health issues, focusing in particular on the NCD-related issues of access to medicines, tobacco control, alcohol control and food and nutrition.

###  Agenda-Setting 

To guide our analysis of the agenda-setting processes that shaped Australia’s negotiating mandate in the TPP, we ex ante drew on Shiffman and Smith’s framework of political prioritisation. According to this framework, global health issues are more likely to receive prioritisation if there is convergence on actor power, ideas, political context, and issue characteristics. First, actor power refers to “the strength of the individuals and organisations concerned with the issue”^28^ including the cohesiveness of advocacy groups, presence of strong leaders, supportive institutions and strong civil society mobilisation. Second, ideas refer to the understandings actors bring to the issue, including the role of framing in generating support (or opposition) to the issue. Successful framing occurs when advocates secure attention to their desired problem and solution through convincing arguments and narratives.^20^ Third, political context refers to the political environments surrounding the policy domain, including policy processes and political structures. Finally, issue characteristics refer to the features of the problem, such as the strength of indicators, severity and presence of effective interventions, which can help or hinder attention to the issue. Using this framework, we sought to investigate the factors shaping attention or neglect of NCD risk factors during Australia’s participation in the TPP. This study is part of a broader project examining agenda-setting and public policy for the social determinants of health equity.

## Key Messages

**Implications for policy makers**

Non-communicable diseases (NCDs) are the cause of more than seventy per cent of deaths globally. Trade and investment agreements facilitate NCD risk factors by increasing the volume of health harmful commodity imports (ie, tobacco, alcohol, ultra-processed foods), as well as local production, manufacturing and distribution of these products through goods and services liberalisation and increased foreign direct investment. Despite recognition of the need to promote greater coherence between health and trade sectors, health remains largely on the periphery of most governments’ trade policies. This study identifies 16 conditions that influence agenda-setting for health in the trade domain. Lessons from success in the areas of access to medicines and tobacco control in Australia could be applied by policy-makers and practitioners to promote greater prioritisation of health on government trade policy agendas.

**Implications for public**

Trade and investment agreements can serve as structural drivers shaping the social determinants of health through a number of pathways, both positive and negative. Greater understanding of the factors that enable attention to health in trade policy agendas can inform improved policy coherence which can help improve outcomes for health.

## Methods

 This article presents results from 25 semi-structured interviews with representatives from key government and non-government actors involved in agenda setting and policy discussions for Australia’s trade objectives during the TPP negotiations.

###  Recruitment

We recruited informants using purposive sampling of representatives for each actor group identified; politicians and their advisors, public servants, academic experts, representatives from industry, and representatives from civil society (see Table 1). Informants were identified from policy submissions to government accessed from the Department of Foreign Affairs and Trade (DFAT) website, from Parliamentary Hansard records and media reporting, and from referral and snowball techniques.

**Table 1:** Informant Position

**Position/Sector**	**Number**
Politicians and political advisors	5
Federal public servants	5
Industry representatives	5
Public interest non-governmental organisations	5
Academic experts	5
Total interviews	25

###  Data Collection 

Prior to interviews, we used a theory-guided process tracing method^29^ to create a timeline of key events during Australia’s participation in the TPP negotiations. Publicly available submissions made by non-government organisations to the Australian government (ie, policy oriented documents expressing their position on the negotiations and what did or did not want the government to agree to) were downloaded from the government website (n = 87), were read and thematically coded using framing analysis and network analysis methods. These analyses are published elsewhere^18,22^and informed the semi-structured interview schedule which focused on actors, ideas, political context, and issue characteristics^28^ (see Table 2). Interview questions were pilot-tested with two experts in trade and investment policy before commencing. Interviews were conducted by the primary investigator (BT) between November 2017 and July 2018, averaged 45-60 minutes duration, and were conducted in English.

**Table 2:** Key Framework Concepts and Associated Questions

**Actors**	Who were the main actors seeking to shape the Australian government’s priorities in the TPP? In your view, which of these actors were influential? Why?
**Ideas**	How were actors positioning their interests and framing their ideas of what the Australian government should prioritize in the TPP? Were there competing ideas?
**Political context**	We have a timeline of key events in this period of 2008-2015 (discuss – what’s missing that was important?) What institutional processes, either formal or informal and inside or outside the trade negotiations did you see as important? Why? Were there turning points or events that shifted the agenda over this time period (this could include changes in government, shifts in public opinion, government reports, external events)?
**Issue characteristics **	Are you aware of any non-market oriented policy priority entering into trade policy? (such as medicines, environment, labour?) Were there differences in attention to different health issues? Why? What role did existing policy or international agreements play, if any?

Abbreviation: TPP, Trans-Pacific Partnership.

###  Analysis

Interviews were recorded, transcribed verbatim and coded using NVivo 11 qualitative data management software. An initial coding scheme was informed by the study questions and Shiffman and Smith^28^ framework. Final codes developed through an iterative process involving inductive analysis of the data with reference to theoretical concepts from the literature, and were discussed by the author team. Analysing informant accounts thematically allowed for identification of core factors and conditions shaping attention or neglect of health risks during Australia’s participation in the TPP negotiations. After identifying, the relative attention to different NCD risk factors (see Results), the strength of each of the factors for each health issue were classified as Weak, Moderate, Strong, Mixed or Unclear. This coding was based on the majority of informants’ views, triangulated with supplementary data including policy actor’s submissions, Parliamentary Hansard records and Australian government export data.

## Results

Interviews indicated that two NCD-related health issues did emerge onto the Australian government’s agenda during the TPP, that of domestic concerns for access to generic medicines and protecting regulatory space for tobacco control. This contrasted with other NCD-related health issues like alcohol control, nutrition and food systems that did not appear to receive attention. Informants identified several factors as shaping the relative attention or neglect of these four health issues, which we classify under the categories of actors, ideas, political context and issue characteristics (see Table 3). We compare and contrast these factors for the below (summarised in Table 4).

**Table 3:** Factors Shaping Attention to NCD-Related Health Risks in Trade Policy

**Actors**	Strength of exporter interests Strength of exporter relationships with government Extent of Trade Minister Health Minister support for health issue Support from other economic actors for health issue Presence of pre-existing health networks in trade policy domain
**Ideas**	Extent of knowledge of health issue in trade negotiations Alignment of health issue with dominant market framing Path-dependency in trade treaty making
**Political context**	Support from major political parties for health issue Exogenous influencing events Advocates use of formal and informal institutional processes inside and outside the negotiations Public support for health issue
**Issue characteristics **	Strength of evidence for health issue (health, economic, trade/health causation, stories) Presence of domestic legislation for health issue Existing international treaties for health issue

Abbreviation: NCD, non-communicable disease.

**Table 4:** Matrix of Conditions and 4 NCD-Related Health Issues in Australia

		**Access to Medicines**	**Tobacco Control**	**Alcohol**	**Nutrition**
Actors	Strength of exporter interests	Weak	Weak	Strong	Strong
	Strength of relationships between industry and government	Unclear	Unclear	Strong	Strong
	Trade Minister and DFAT support for health issue	Strong	Strong	Weak	Weak
	Health Minister and Department of Health support for health issue	Strong	Strong	Weak	Weak
	Productivity Commission support for health issue	Strong	Strong	Unclear	Unclear
	Extent of pre-existing health networks in trade domain	Strong	Weak	Weak	Weak
Ideas	Alignment of health issue with dominant market framing	Strong	Weak	Weak	Weak
	Path dependency of trade treaties	Mixed	Mixed	Strong	Moderate
	Knowledge of health issue during TPP negotiations	Moderate	Strong	Weak	Weak
Political context	Support of political parties for health issue	Strong	Strong	Weak	Weak
	Exogenous events during TPP negotiations	Weak	Strong	Weak	Weak
	Advocates use of formal and informal institutional processes inside and outside the negotiations	Strong	Unclear	Weak	Weak
	Public support for health issue	Strong	Strong	Weak	Weak
Issue characteristics	Strength of evidence for health issue	Strong	Strong	Mixed	Mixed
	Presence of domestic legislation on health issue	Strong	Strong	Mixed	Weak
	Existing international treaty on health issue	Strong	Strong	Weak	Weak

Abbreviations: NCD, non-communicable disease; TPP, Trans-Pacific Partnership; DFAT, Department of Foreign Affairs and Trade.

###  Actors

 Six factors appeared to influence Australian actors’ attention to NCD-related health issues during the TPP negotiations.

####  1. Strength of Export Interests

Exporter interests in the particular NCD issue domain were seen as a key factor influencing government attention or neglect of public health arguments in the trade domain. In the case of tobacco, there was no exporter interest because production and manufacturing in Australia have been progressively scaled back over time (and ceased altogether in 2016), while a suite of tobacco control measures have been progressively implemented including tobacco plain packaging legislation announced during the TPP negotiations. In the case of medicines, Australia had defensive interests as an IP-importing nation with a generic medicine industry and low pharmaceutical exports (ie, strong import interest). Public health advocates were joined by the Australian generic medicines industry and industry actors in other sectors like telecommunications in opposing the extension of IP.^30,31^ As one industry informant noted:

*“Australia is an IP importer, so it’s not in our interest to extend the terms of IP where that may increase the cost to the Australian economy”* (industry).

 Furthermore, other powerful industry bodies did not want to see their own export interests suffering by having the agreement stalled over debates concerning extended IP provisions and access to medicines, or concerns over protecting regulatory space for health. As one industry association representative suggested:

*“… so investor state dispute settlement clauses are something we’re very aware of because it’s been a significant challenge in the TPP and other agreements... the biologics issue in the TPP on intellectual property too... we look at it and go ‘what else is going to stop this agreement coming into place?’ and those issues regularly come up…. If the agreements isn’t going to get signed because of these well we really encourage the government to sort that”* (industry).

 In contrast, informants view Australia’s offensive interests (ie, strong export interests) in exporting alcohol and sugar as barriers for generating attention to the health impacts in the TPP negotiations:

“ *We had some ugliness in the WTO when we’re trying to do plain packaging on tobacco, and Australia is in the WTO criticising Thailand for its labelling measures, and we had to do a lot of work to get DFAT to pull back from that... but in the TPP we didn’t get far on alcohol labelling because DFAT saw market access... as being more important than a public health principle” *(public servant, health).

####  2. Strength of Relationships Between Industry and Government

 Strong relationships between exporters and government officials played a major role in developing Australia’s objectives in the TPP. Industry informants were clear about the influence they had in trade agenda-setting:

*“ So they’ll [government] ask us, ‘there’s a free trade agreement negotiation on... what are your priorities? Are there any issues your members are concerned about?’ and we will have input where we identify particular issues our members have raised”* (industry).

 As government trade officials similarly noted:

*“ Certainly the exporters have a very concrete and specific interest. And the agreements are for them. And so you need to ensure that it’s actually going to serve their interests basically” *(public servant, trade).

 In some instances, exporting industry actors reported being asked to provide information to assist government negotiators, including draft texts and arguments:

*“They’re very interested in information that they can use to make the argument…Sometimes they ask for that sort of information …. ‘you’ve got to give us some arguments that will help us persuade them,’ which are the arguments a negotiator would presumably use in a room with a counter party”* (industry).

 In contrast, a majority of public interest and public health oriented informants reported weaker connections and relationships with government overall, and weaker engagement on areas of Australia’s offensive interests:

*“Initially we tried to talk to DFAT and they wouldn’t even talk to us... it took a couple of years for them to actually talk to us”* (civil society).

*“It was really important to be able to have discussions with particular negotiators... but it was difficult to get any useful information... particularly on issues where Australia had offensive interests, like alcohol and food” *(academic).

####  3. Trade Minister Support for NCD Issue

 A third factor that appeared to shape attention or neglect of an NCD issue in trade policy was the extent of support for the issue from the Trade Minister in Australia, the core executive of the government is a committee of government Ministers (known as the Cabinet), chaired by the Prime Minister. The Trade Minister is a member of the Cabinet and administers the trade portfolio including overseeing and signing off on Australia’s trade agreements. The Trade Minister sets the negotiating mandate for government negotiators in the DFAT who act on behalf of the government in negotiating trade agreements.

 Despite a change of government during TPP negotiations, there was a clear mandate for attention to issues around access to medicines and tobacco control from successive Trade Ministers:

*“…the issue that we were dealing with being tobacco was one where the Trade Minister was on side and very aware of it and really the discussions were ‘ yes we’re making sure that this [the TPP] wouldn’t do anything that would affect our ability to do that’ [implement tobacco plain packaging]”* (politician).

*“ So our mandate in the pharmaceutical area was very clear and it didn’t change between governments... where pharmaceuticals was discussed we had a number of meetings with DFAT and they were consulting with other groups... it was very collegial”* (public servant, health).

####  4. Health Minister Support for NCD Issue

 Leadership in the Federal Department of Health was seen as a key factor that enabled greater attention to tobacco control during the TPP negotiations. Informants reported a strong position on tobacco control from successive Health Ministers (who are also members of the Cabinet), but less attention to alcohol, food and nutrition:

*“She’d [Nicola Roxon, Health Minister 2007-2011] made an enemy of the tobacco industry and she just wasn’t willing to take more heat... she was going strong on tobacco [but] going into the TPP really not up to making a huge splash on alcohol...” *(public servant, health).

 There was also an absence of other government voices outside of the trade and economic sectors. As one informant noted of the 2013 shift of Australia’s government development aid into DFAT:

*“We’ve lost AusAID as a separate voice, so at least they would have been a voice saying ‘you’ve got to think about the development needs of countries and we can’t just make them all incredibly unhealthy by dumping our alcohol and lamb flaps onto them.’ You need other voices [than Health] to be making the equity case.” *(public servant, health).

####  5. Other Economic Actors Support for NCD Issue

 Attention to the issue of access to medicines during the TPP was also bolstered with support from other economic actors, in particular the Productivity Commission, an independent government advisory body. Tasked with conducting an inquiry into bilateral and regional trade agreements (BRTAs), the Commission released its report in 2010, which offered economic analysis and arguments against elevating IP and against the inclusion of ISDS provisions. It recommended that the Australian government:

*“Not seek to include intellectual property provisions in Australia’s BRTAs as an ordinary matter of course (Productivity Commission, 2010, p. xxx ii) and... avoid the inclusion of ISDS provisions in BRTAs that grant foreign investors in Australia substantive or procedural rights greater than those enjoyed by Australian investors” *(p. xxxviii).^32^

 The assessments provided an economic argument that was influential on the centre-left Labor party (in government 2007-2013) adopting a “no ISDS” position in the TPP negotiations:

*“We did adopt a position [against ISDS] that was based on what the Productivity Commission was indicating that by giving up jurisdiction we could have our environmental and our health objectives compromised by not being able to carry out genuine domestic public policy”* (politician).

 This position, however, did not survive a change of government in 2013, with the election of the conservative LNP coalition.

####  6. Presence of Pre-existing NCD Issue Networks in Trade Policy

 Many informants reported that their understanding and attention to the issues of access to medicines and ISDS in the TPP negotiations were informed by the legacy of the US-Australia Free Trade Agreement (AUSFTA) negotiated a few years earlier:

*“It helped that there was an existing body of work on the issue already [on IP in trade and access to medicines]. So really clear cut, easy to understand concerns that the trade negotiations raised” *(academic).

*“With [AUSFTA] we’d had this huge debate about ISDS and in the end ISDS wasn’t included. But we knew the Americans would be putting it up in the TPP... so from the beginning we said ‘we don’t want ISDS and we don’t want additional medicine monopolies in this agreement’”* (civil society).

In contrast, there were no existing networks focused on trade agreements and alcohol, nutrition or food policy in Australia, with little pre-existing awareness of connections with trade policy in government: “ *at the time governments weren’t so active in tobacco or alcohol law or food policy, so we kind of just missed those issues*” (public servant, health).

###  Ideas

 In addition to actors, we identified three ideational factors that shaped NCD-related health issues for Australia’s participation in the TPP: knowledge of the potential trade-related impacts of the health issue; alignment of the health issue with the dominant market framing; and path dependency of existing trade treaties.

####  1. Knowledge of Potential Trade-Related Impact on the Health Issue

 Policy actors’ knowledge of the status of the TPP negotiations and trade provisions that could affect an NCD health issue was a key driver in their attention and associated advocacy or engagement:

*“There was awareness that in the TPP the US was seeking a set of more ambitious outcomes in some areas of pharmaceuticals... so there’s some policy areas, including public health, where negotiators know that they’re sensitive and you need to ensure that there are ways to accommodate those interests”* (public servant, trade).

 However, because the negotiations were conducted between governments outside of the public sphere, knowledge of issues under negotiation for many non-government public health actors was informed by leaked texts, which was identified by informants as a key factor for providing knowledge of potential risks:

*“The only decent leak was the chapter on intellectual property and that was invaluable because we were able to demonstrate the sorts of things that were wrong with the agreement, and it gave people a sense of what was going on... so stronger patents for medicines...”* (politician).

*“The leaks were very, very significant in terms of what they enabled people to do. In terms of getting the media attention to the issues. In terms of being able to present analysis, in terms of being able to focus on specifics within the agreement that could be bad for the public” *(academic).

 Leaks of intellectual property chapters provided greater knowledge of potential risks for access to medicines, however many chapters were not leaked, which constrained knowledge of potential risks for other health issues.

####  2. Alignment of Health Issue With Dominant Market Framing

 Frame alignment was another key ideational factor for enabling greater attention to access to medicines over alcohol, food, and tobacco. For many informants, the dominance of market and neoliberal ideas in the trade domain were particularly constraining for advancing a broad social determinants of health agenda:

*“I think that 20 years of neoliberal economics have kind of poisoned a lot of the communication channels, where if you show evidence that the deal won’t increase GDP [gross domestic product] despite what the government says, people find it hard to believe some of that”* (union).

 Informants reflected that the framing of access to generic medicines aligned with the dominant market language of promoting greater competition and access to goods, whereas arguments concerning alcohol control and unhealthy food did not align so easily and instead had strong offensive industry interests arguing against public health concerns:

*“ So medicines can reduce access to goods, but the issue of exposing someone to something bad is harder to defend... you know, it’s ‘you’re forcing a product on someone’ versus depriving someone of a product”* (politician).

*“We would always resist having anything you consider to be a core quality attribute regulated. So nutritional content is a quality attribute rather than a pest or disease related issue [and], we would generally [resist] putting it into a trade agreement” *(public servant, agriculture).

####  3. Path Dependency of Trade Treaties

 Path dependency of previous trade treaties was another ideational factor shaping government’s willingness to consider potential NCD implications of TPP treaty text. Informants reflected that what Australia had already agreed to in the World Trade Organization (WTO) and existing agreements shaped the approach to negotiations in the TPP:

*“ So the biggest determinant of what ended up in the TPP from an Australian perspective was what we’d already agreed to elsewhere”* (academic).

*“I know that there was a lot of concerns in TPP committee meetings about nutritional labelling, for example in Chile, Mexico, where they want to have a big red thing or big black thing saying high in fat and salt and things like that. And a lot of countries were bringing concerns saying it’s not consistent with the WTO”* (public servant, health).

*“If it’s already there, it’s very hard to get any changes or concessions in the new text... we lost a battle over a decade ago with the US free trade agreement [on copyright]… it makes it harder for us to go out there guns blazing [on the TPP] because we’ve already lost that battle”* (public interest organisation).

###  Political Context

 In addition to actors and ideas, 4 key political factors appeared to shape attention or neglect of NCD issues: support from major political parties; exogenous influencing events advocates use of formal and informal institutional processes inside and outside the negotiations; and the extent of public support.

####  1. Support of Political Parties in Government

The extent of major political party support for an NCD issue was a key political factor shaping attention or neglect in the TPP negotiations. Both major political parties in Australia were in government during the TPP negotiations. Both major parties supported the TPP negotiations, and gave statements in support of ensuring access to medicines. Neither party championed alcohol or food and nutrition issues. There was a clear difference, however, in their approach to ISDS. During the ALP’s period in government (2003-2013) there was a clear shift to ‘no ISDS’ emphasis in TPP negotiations, while the LNP in 2013 re-introduced negotiations on ISDS. Informants identified this shift as significant, with “ *Australia ultimately agreeing to include ISDS in the FTA,*” (public servant). More broadly, informants considered that certain NCD issues were shaped by differing ideologies and broader electoral concerns:

*“This colour of government [conservative Liberal-National coalition], they don’t want to regulate food, they don’t want to do that stuff, so they’re quite happy to see that traded away... so when there’s a government that’s just not going to regulate anything, the advocacy’s got to be a bit cleverer in a way. It’s no use shouting public health from the rooftop, because they’re not going to listen”* (public servant).

*“If we did a sugar tax, we’d do it like we do tobacco tax, there would be an excise so everyone’s on the same footing. But governments would lose all the seats in the sugar producing areas of Queensland, right. So there’s a domestic political imperative that’s hard to get over” *(public servant).

####  2. Exogenous Influencing Events

 A key factor identified by informants for shaping greater attention to tobacco control during TPP negotiations was the litigation by tobacco firm Phillip Morris against the Australian government over its tobacco plain packaging legislation. Although this litigation was external to the TPP negotiations, informants reported that it drew attention to the potential risks of ISDS for public health, influencing Australia’s negotiating mandate around protections for public health in the TPP:

*“When those issues appeared in Cabinet the rationale for not going down the path of ISDS was the Phillip Morris example”* (politician).

*“…everyone was aware of that, all the negotiators were aware of that” *(public servant).

*“…[it] was ongoing throughout the TPP negotiations [raising] questions around... whether something like the TPP could prevent other countries from adopting things like plain packaging”* (academic).

 In contrast, there were no obvious external influencing events in shaping attention to other NCD issues during the negotiations.

####  3. Advocates Use of Formal and Informal Institutional Processes Inside and Outside the Negotiations

 Overall, industry actors were more engaged and supportive of government processes for consultation and engagement than public health and public interest advocates we spoke to:

*“They hold public consultations which are notable for their lack of information and the lack of openness to discussion” *(academic).

*“I’ve been in more industry focused ones [consultations], where you’re asked to reflect a particular industry view in more depth”* (industry).

 Informants also spoke of the importance of informal processes for engagement inside the trade portfolio, such as informal conversation with negotiators in the corridors alongside trade rounds. Generally, public interest advocates noted that discussion was more open and informative on issues where Australia’s interests aligned with their interests, suggesting that Australia’s interests shaped non-governmental organisation and experts access to the informal processes. Finally, informants also highlighted the importance of using processes outside the negotiations such as lobbying parliamentarians (informal) and using parliamentary committees (formal) to raise public health concerns. For many public interest informants, a dual strategy was needed to make use of mechanisms inside the trade portfolio and an outside game of strategy and campaigning. Of the NCD related health issues, the issue of access to medicines appeared to receive the most attention from public health actors who used informal and formal processes both inside and outside the negotiations.

####  4. Public Support

 Informants noted strong public support for Australia’s public health system, Pharmaceutical Benefits Scheme, and tobacco control measures as influencing successive government’s defensive attention to these issue in the negotiations. In contrast, weaker evidence of public support for alcohol control or nutrition labelling suggested less political incentive to focus on these issues:

*“Our Medicare system and our pharmaceutical benefits system are very much like the holy grail. In Australia, you can’t touch it”* (trade union representative).

*“Where there’s a high level of market acceptance for something bad currently it is harder to make those cases... for example be it sugary drinks or alcohol, they’re so embedded in our culture, despite the harm they’re doing, that it will take a long time to actually undo that” *(politician).

###  Issue Characteristics

 Informants identified differences in the strength of evidence and presence or absence of domestic legislation and international treaties in shaping diverging government attention to NCD-related health issues in the TPP negotiations.

####  1. Strength of Evidence 

 The strength of health evidence was viewed as an important factor:

*“We haven’t built the evidence on nutrition and obesity as well as [on] tobacco, it’s kind of more obvious, here’s a product that you consume that kills you... nutrition, it’s ubiquitous and it needs to be all levels of government”* (public servant).

*“We’re struggling with half of the world not being [fed] enough and the other half being over-fed and it’s difficult to find some internationally consistent approach, whereas it’s not that difficult tobacco-wise and... alcohol is somewhere in the middle of that” *(politician).

 The persuasiveness of economic evidence was also clear, with informants pointing to the influence of costings illustrating the potential impacts of trade agreements for access to medicines or for public health regulation:

*“[With tobacco] people understand the cost in human welfare and financial costs in healthcare so any economic damage to the relevant sector is outweighed by the economic cost to the people and to the taxpayer”* (industry).

*“Trade policy is all about economics, or at least nominally... What really gets traction with trade negotiators, with politicians and also what grabs the public’s attention is the economic costs” *(academic).

 Evidence of the causal link between a trade provision and its potential impact on health was also viewed as important. Informants noted clearer causal links between an IP mechanism that delays generic entry of pharmaceuticals, but less causal clarity around trade liberalisation of food and associated morbidity and mortality:

*“It’s so much more direct, because everyone understands that medicines can be expensive. If they are too expensive, then you cannot take it. If you cannot take it, you get sick. If you stay sick, you can die... when you start to talk about how import rules around alcohol increase access or the availability to sell alcohol, and how evidence shows the availability of alcohol increases consumption, the pathway is a bit more distant and requires a bit more understanding to draw that out”* (academic).

*“To be able to influence the outcomes you need to pinpoint what needs to be changed in the agreement. I think there’s a long way to go in the food area in terms of being able to say ‘if we change this, if we tweak that, well get a better outcome,’ it’s not easy” *(academic).

 Related to understanding the causal connections was trade negotiators’ desire for greater specificity on public health concerns and asks:

*“Sometimes submissions raise concerns but it’s not clear how the trade agreements would actually bring about the effect they’re worried about... and so its [not] uncommon for submission to claim, you know that trade agreements will prevent A,B,C but without being able to show how that would be the case…” *(public servant).

Whether evidence has already been accepted at WTO also appeared to influence government, with some government informants preferring evidence that had been accepted at WTO. One government informant, for example, argued that Australia’s objectives to include measures to protect biosecurity and plants in the TPP were based on “ *legitimate scientifically based requirements to keep pests and diseases out*” acknowledged at WTO. In contrast, proposals from civil society for the inclusion of evidence-based animal welfare standards were not influential for government because “ *they’re not part of WTO*” (public servant, agriculture).

 Related to the strength of evidence was the ease in which evidence could be used in narratives and framing of the policy issue. The use of personal stories as a source of evidence was highlighted by many informants as influencing public debate around the TPP:

*“There was a whole case with a woman who was advocating for better access for biologics and she was trying to get better cancer treatment, and so being able to give her personal story of why this type of drug is a lifesaver and she couldn’t afford it without a generic. That kind of connection, that real life story makes it accessible” *(academic).

####  2. Presence of Domestic Legislation

 The presence of existing domestic legislation for tobacco control (ie, tobacco plain packaging), and public-subsidised medicines (Medicare and the Pharmaceutical Benefits Scheme) were identified as important in shaping attention to these issues during TPP negotiations.

*“Tobacco plain packaging only got up because it got up as a health issue in Australia first... the formula seemed to be that there was already a campaign in Australia”* (politician).

 This was contrasted with a potential barrier in the form of trying to protect future policy space not yet enforced domestically:

*Access to medicines and the right to uphold our own laws are kind of things we were protecting. Whereas the other *issues are prospective things [eg, mandatory alcohol labelling] *...* we haven’t done them yet. So they have a different relationship to the public interest test” (politician).

*“We don’t have a domestic framework that defines the public interest in any real terms when it comes to food and nutrition... we’ve already got a framework around access to drugs being in our public interest... But when it comes to food, we haven’t got a framework around that”* (politician).

####  3. Existing International Treaty

Finally, informants reported the *Framework Convention on Tobacco Control* (FCTC) to which Australia is a signatory as influential in shaping and enabling defence of Australia’s tobacco control efforts, while the WTO *Doha Declaration on TRIPS and Public Health* provided strong statements to ensure that trade agreements do not weaken access to medicines:

*“[the FCTC] provided a really important normative counterpoint about the responsibilities of states to move forward in terms of tobacco control, and so the fact we didn’t have an agreement like that for issues like alcohol and food made it much more difficult to get traction on those issues” *(academic).

 Opportunities for using international health organisations in the future was also highlighted as providing a venue for elevating health above trade:

*“We’ve had a couple of fights in the WHO [World Health Organization] context about guidelines on sugar or on dairy where those industries have rung up the Trade Ministry and go the Minister to complain to the [Health] Minister… and what’s good is that we will always defend the science and DFAT won’t get to overrule that… so the WHO setting is really important for setting those norms and standards that we can get applied in another setting”* (public servant, health).

###  Political Dynamics Inside the Trans-Pacific Partnership Negotiations

While we have shown that the issue of access to medicines was on the Australian government’s agenda and negotiating mandate during the TPP, it is important to note that the outcome of the negotiations was mixed for the participating countries. In the final TPP text, several low and middle-income countries agreed to elevated levels of IP beyond their existing domestic laws, raising concerns of the impact to access to generics in those countries.^14^For Australia, the outcome did not lead to a change in existing IP law, but did lock in existing IP commitments and, for the first time, included commitments on protecting IP for biologic medicines. On the one hand:

“ *Australia was kind of leading the case [on medicines] for a whole lot of other countries that had the same approach and concerns and the US and Japan were aligned on the other side... but [Australia] was appreciated because access to medicines is such a crucial defensive issue for every country” *(public servant, health).

 While on the other:

*“It would be preferable to not have any agreement about biologics [but] at the end of the day after three or four days and nights of negotiation and wordsmithing and going backwards and forwards [with] Ministers there involved in this and wanting to conclude an agreement... it is a compromise [because] you know, the US was going to have something on biologics in this agreement” *(public servant, health).

The issue of ISDS was also controversial inside the TPP negotiations. The final text of the TPP investment chapter did include a specific option for countries to deny the use of ISDS for claims applying to tobacco control measures, known as the “tobacco carve-out.”^33^ While this carve-out was widely praised by tobacco control groups, informants’ views of the political dynamics inside the TPP negotiations were more critical:

*“Australia was arguing heavily for a broad public health carve out and internal US politics led to a specific tobacco carve out being introduced… and there was kind of a sense ‘well you public health people got what you need now, so chuck the rest out’ and that’s a disadvantage for alcohol and food and other areas of public health where governments might want to intervene... and you know, tobacco exceptionalism won the day” *(public servant, health).

 One informant further argued that the debate over ISDS and tobacco in the TPP created problems outside the negotiations for countries looking to implement tobacco control measures:

*“A lot of the narrative about all the ways in which trade and investment agreements prevent countries from implementing tobacco control measures, I think was really over exaggerated... because they’re in WTO agreements, they’re in a whole range of international agreements and those weren’t going to disappear.”* (civil society).

 The conflict over ISDS and tobacco was also viewed as a reason to not go down the path of a sugar tax in Australia by one politician:

*“There’s a lot of discussion with a sugar tax or something like that... but then how do you deal with that when you have your own industry of sugar and you’re also an international industry of sugar. And you have a free trade agreement. So how do those complexities get read through? And the answer is, they don’t, because the tobacco experience was they just take you to the ISDS and the best result is you spend millions of dollars to have the case dismissed” *(Politician).

## Discussion

 Our analysis has identified 16 factors shaping attention or neglect of NCD-related health risks in trade policy in Australia. We found evidence from informants that two health issues, domestic concerns for access to medicines and protecting regulatory space for tobacco control, did emerge onto the government’s negotiating mandate. This contrasted with other NCD related risk factors, alcohol control and nutrition, which a majority of informants identified as not receiving political attention. In this section, we reflect on the findings for NCD advocacy and promoting greater prioritisation of health in trade policy.

First, we found that Australia’s export interests was a key factor in whether the government would seriously consider potential NCD risks in the trade negotiation. This finding provides evidence to support recent arguments that NCD policy options need to give attention to the supply side of health harmful products.^34^ Measures that would encourage industry to transition to healthier products, such as using a ‘Just Transitions’ framework like in the climate and energy space,^35^ could be used in the NCD space.

 Second, our finding on the importance of strong political leadership suggests that Health Ministers and health officials can play an important role in drawing attention to health impacts of trade deals, but it is important that they have the knowledge and trade literacy. Building on the work by WHO and the McCabe Centre, capacity building and training of Health Ministers and officials on the potential health risks for NCDs in trade policy is vital. In addition, the presence of existing informal networks around a health issue was found to be important for providing attention to health concerns at the start of a trade agreement negotiation. Civil society organisations and professional health associations play a key role in monitoring trade agreements, as was identified for the issue of access to medicines in our case study. This monitoring capacity, however, also requires greater trade literacy amongst the health community.

 Third, the potential wide ranging impacts of trade agreements on the social determinants of health, including environment, employment, human rights and social sectors, illustrates opportunities for a broader coalition of public interest actors (such as environmental and human rights non-governmental organisations), and invoking the normative power of related treaties (eg, the United Nations Convention on the Rights of the Child, International Covenant on Civil and Political Rights and the International Covenant on Economic, Social and Cultural Rights). We found evidence of a public interest network in Australia comprising trade unions, not for profits, community organisations and a select few public health organisations. There could also be much greater engagement by NCD focused and peak public health bodies in this network. In addition, we found that support from economic actors was useful for promoting health issues. Public interest and public health actors in the trade domain could widen their coalitions to include more supporters from outside the health domain to bolster support for social and health goals.

Fourth, government processes for negotiating trade deals need to be much more transparent and consultative. Informants reported a reliance on leaked text during the TPP to analyse the potential health impacts of proposed intellectual property provisions for access to medicines. Other research finds that even government health officials (such as in Malaysia) have reported relying on leak texts to assess potential health impacts.^23^ Models such as the European Union’s policy to publicly release negotiating texts^36^ are a start to greater transparency. However, transparency means little without meaningful consultation. The analysis suggests that institutional changes are urgently needed to formally include health representation, and echoes calls for greater transparency, accountability, participation, integrity and capacity in the governance of trade policy.^17^ Thailand has been a leader in this regard with its interdepartmental *International Trade and Health Programme* to generate evidence-based analyses of the potential health impacts of trade negotiations for government.^37^ Thailand’s former Constitution included requirements for parliamentary approval of trade negotiation frameworks but these constitutional protections were unfortunately after the 2014 coup d’état. Institutional changes that could be implemented to promote health include formal processes for Health Minister – Trade Minister dialogue on potential impacts of trade deals, trade and health officials’ consultation on all matters that can affect health regulation throughout the negotiations, publishing of trade texts, and mandatory health impact assessments during the negotiations and before the final text is signed.

Fifth, we found that framing of health issues was also a key factor for generating attention. In particular, economic frames and arguments were useful for promoting attention to the need to ensure access to medicines. This economic framing could be used for drawing attention to the costs of NCD harm on society and the economy. For example, whereas some politicians appeared hesitant to support public health measures due to concerns over multi-million dollar ISDS cases (see results above), the multibillion dollar costs of NCDs on healthcare and lost productivity far outweigh these costs. Furthermore, the potential costs of ISDS for government on public health suggest the need to remove ISDS from trade deals, not avoid public health regulation. It is also worth noting that the use of economic framing and cost-effective arguments could be limiting because it ignores the wider social determinants and privileges economics and private enterprise over social goods.^18^ The result is an ethical and argumentative trade-off that needs careful consideration by public health advocates.

Sixth, our analysis points to the need for further studies to collect the evidence on the causal connections between NCD risk products and trade deals, and for greater advocacy and lobbying on the part of civil society. Informants noted that having existing domestic health policies and legislation, such as Australia’s publicly subsidised prescription medicine scheme, and international health treaties, such as the FCTC, were useful for making the health case in the trade domain. The lack of a national nutrition policy in Australia was particularly evident as a gap, suggesting that national health policy development is important for defending health interests in trade policy-making. Furthermore, it is noteworthy that no informants identified the WHO Global Strategy on Alcohol or existing national alcohol policies, suggesting that a binding treaty such as a proposed Framework Convention on Alcohol Control^38^ or other framework legislation on NCDs^39^ might have greater normative weight in a trade context than WHO standards. There is scope here for public health and NCD advocates to make use of domestic and global health measures on tackling alcohol harm and junk nutrition to promote greater attention to these issues in trade negotiations.

These findings may also have applicability beyond the trade domain for thinking about mechanisms for prioritising health in government policy areas outside the medical-health sector. We note that the Shiffman and Smith’s framework^28^ that guided our analysis used emerged from a study of global maternal health policy, where policy actors understand the health remit but are torn between competing health issues for prioritisation. Our analysis has revealed sixteen particular conditions to the trade domain that are likely to have applicability to other policy areas in the commercial determinants of health. Further health research could apply this framework to cases in the social and commercial determinants of health to identify the range of conditions that enable or constrain health, and thus reveal potential strategies that generate successful prioritisation of health in economic sectors. Further application of this framework of other trade agreements and in other countries could also generate additional valuable lessons on specific health topics, and specific country context. Finally, this public health analysis offers the political economy field another lens on the implications of policies, providing a broader view of policy purpose. The analysis provokes international political economy scholars to ask ‘for what purpose’ when deliberating the regimes and institutions associated with trade and other structural factors.

## Conclusion

 Drawing on a qualitative case study of Australia’s participation in the TPP, this article provides policy actors’ insights into the factors that can enable or constrain attention to NCD risk factors in trade negotiations. Interviews indicated that domestic concerns for protecting regulatory space for access to generic medicines and tobacco control emerged onto the Australian government’s negotiating mandate. This contrasted with other health issues like alcohol control and ultra-processed foods that did not appear to receive attention. Applying a framework of political prioritisation, we identify sixteen factors which shaped varying attention to these issues. These findings aid our understanding of the factors that can enable or constrain attention to health in trade policy-making, and offer insights for potential pathways to elevate greater attention to health in the future.

## Ethical issues

 Ethics approval for this study was received from Flinders University Social and Behavioural Research Ethics Committee (Project number 6786) and The Australia National University Human Research Ethics Committee (Protocol 2015/243).

## Competing interests

 This study was funded by a grant by the Australian National Health and Medical Research Council (NHMRC). The NHMRC had no role in the conduct or writing up of this study. BT did not receive any grants during this study and has no competing interests, but the NHMRC funded this study - which is already stated in the funding disclosure section.

## Authors’ contributions

 Conception and design: BT, SF, FB. Acquisition of data: BT. Analysis and interpretation of data: BT, SF, AS, FB, RL. Drafting of the manuscript: BT. Critical revision of the manuscript for important intellectual content: BT, SF, AS, FB, RL. Statistical analysis: BT. Obtaining funding: SF, FB, RL. Administrative, technical, or material support: BT. Supervision: SF, FB, RL.

## Funding

 This work was supported by the Australian NHMRC Centre of Research Excellence on the Social Determinants of Health Equity: Policy research on the social determinants of health equity (APP1078046). The NHMRC had no role in the conduct of this research.

## Authors’ affiliations

^1^School of Regulation and Global Governance, College of Asia and the Pacific, Australian National University, Canberra, ACT, Australia. ^2^Southgate Institute for Health, Society and Equity, Department of Public Health, Flinders University, Adelaide, SA, Australia. ^3^Institute of Population Health, University of Ottawa, Ottawa, ON, Canada.
